# A Quantitative Study on Determinants of COVID-19 Vaccine Uptake in a Mandatory Vaccination Workplace Setting in South Africa

**DOI:** 10.3390/ijerph22060929

**Published:** 2025-06-12

**Authors:** Dhirisha Naidoo, Bernard Hope Taderera

**Affiliations:** 1 BroadReach Health Development, Bridgeway Precinct, Century City, Cape Town 7441, South Africa; 2 Department of Environmental Health, Faculty of Health Sciences, University of Johannesburg, Johannesburg 2028, South Africa; btaderera@uj.ac.za

**Keywords:** COVID-19, determinants of COVID-19 vaccination, mandatory vaccine policy, health workers, South Africa

## Abstract

Coronavirus disease 2019 (COVID-19) resulted in significant morbidity and mortality globally. Despite the efficacy of COVID-19 vaccines in reducing morbidity and mortality, uptake in South Africa was sub-optimal due to a number of factors which remain not fully understood, particularly in mandatory vaccination workplace settings. This quantitative, cross-sectional study aimed to understand determinants of COVID-19 vaccination uptake among clinical and non-clinical workers, aged 18 years and older, employed at a large organisation with a mandatory workplace COVID-19 vaccination policy in South Africa. Workers completed a one-off, self-administered, online questionnaire that explored determinants of COVID-19 vaccination, barriers and enablers to accessing vaccines, and perspectives regarding the mandatory workplace vaccine policy. Among the 88 workers enrolled in the study, the frequent reasons for COVID-19 vaccination included preventing the spread of COVID-19 (71%, *n* = 62), fear of contracting COVID-19 (64%, *n* = 56), protecting colleagues and patients (63%, *n* = 55), and the mandatory workplace policy (65%, *n* = 57). Just under two-thirds of workers (63%, *n* = 55) were supportive/very supportive of the mandatory COVID-19 vaccine policy. Reasons for support included the fact that vaccination would create a safer work environment, protecting oneself/others from acquiring COVID-19, and receiving support from their employer. Only 15% (*n* = 13) of workers were not supportive/against the policy. The findings of this study could inform occupational health policy and counselling and support in workplaces in future pandemics.

## 1. Introduction

Coronavirus, also known as severe acute respiratory syndrome coronavirus 2 (SARS-CoV-2), is a virus that causes coronavirus disease (COVID-19) [1]. It is spread through droplets and virus particles that are released into the air [2]. The first case of COVID-19 was discovered in China in December 2019. Thereafter, COVID-19 rapidly spread throughout the world. The World Health Organization (WHO) declared COVID-19 a pandemic on the 11 March 2020 [1]. In South Africa, the first case of COVID-19 was discovered on 5 March 2020, and subsequently, more than 4 million cases of COVID-19 were documented, resulting in significant morbidity and mortality, including >100,000 deaths [3]. Several countries, including South Africa, initiated national lockdowns to curb the spread of COVID-19 [4]. More than a third of the global population had been exposed to a form of restriction of movement by April 2020 [5]. The South African Government implemented measures to reduce COVID-19 transmission in the absence of a vaccine, which included multiple preventative interventions [6]. A national state of disaster was declared in South Africa in March 2020, followed by a nationwide lockdown. The country experienced several significant waves of infection.

Behaviours around vaccines are identified as being complex, and there are many determinants around decisions to take vaccinations [7]. Vaccine acceptance rates from a systematic review from 33 countries ranged from 24% in Kuwait to 97% in Ecuador for the general population, and amongst health care workers, it ranged from 28% in the Democratic Republic of Congo to 78% in Israel [8]. South Africa had a target to vaccinate 70 percent of the adult population against COVID-19 by the end of 2021; however, by May 2022, only 45 percent of the total adult population were vaccinated [9]. Vaccination uptake may be influenced by several factors such as access, social norms, personal beliefs/attitudes, myths/misconceptions, and other factors [10]. Furthermore, vaccine uptake may differ between certain settings and countries. Understanding the motivations for COVID-19 vaccination among workers in high vaccination settings is important, as it can assist in creating effective strategies to increase vaccination uptake and coverage for current and future epidemics. An additional consideration for COVID-19 vaccines among workers is the implementation of mandatory vaccination policies or mandates. This remains a complex and controversial topic, and specifically for healthcare workers, individual rights versus ethical and professional obligations are relevant [11]. In Sub Saharan Africa, 15 studies with about 7500 participants observed a pooled prevalence of COVID-19 vaccination hesitancy among health care workers of 46% (CI 0.38–0.54) and concluded that there was a need to better understand obstacles health care workers faced in vaccinating as they have the potential to influence the broader public [12]. Vaccination mandate studies across different countries have shown rapid uptake initially, with reduced uptake over time, and high vaccination coverage has been shown to be associated with reducing the health and economic impacts of the epidemic [13].

In view of the sub-optimal COVID-19 vaccine uptake in South Africa and limited data on reasons for vaccine uptake in mandatory workplace settings, this study aimed to assess the motivations for COVID-19 vaccination among workers employed at a large organisation in South Africa with a mandatory COVID-19 vaccination policy, where vaccine uptake was 99%. This study also explored barriers and enablers to COVID-19 vaccination and worker perspectives on the mandatory workplace COVID-19 vaccine policy that the organisation had implemented. In this regard, the hypothesis of this study was that a mandatory vaccination work environment results in higher vaccination uptake and acceptance amongst employees, which in turn may help make healthy workplaces functional and contribute positively towards intervening against a pandemic.

## 2. Materials and Methods

### 2.1. Study Design and Population

A quantitative cross-sectional study was conducted from September to October 2024 among workers in a large organisation that supports the South African Department of Health to provide direct service delivery and technical assistance for the Human Immunodeficiency Virus (HIV) and Tuberculosis (TB) programme in the public sector. Workers included clinical and non-clinical staff. The clinical staff included nurses, doctors, pharmacists who provide direct services, laboratory technicians who provide direct support at facilities, and social workers who directly support patients at the facility level. The non-clinical staff included lay staff such as lay counsellors who are responsible for HIV testing services and case managers who are responsible for tracking and tracing clients who are due for appointments and who have missed appointments. Data staff are responsible for capturing patient-level data at facilities, and multi-district service staff include administrative, human resources, finance, contract grants, and compliance and strategic information staff who are responsible for reporting. Whilst this study was conducted in South Africa, staff were based across different provinces in the country, with most of the staff who offer direct services residing in Mpumalanga province and the rest of the staff residing in the Gauteng, KwaZulu-Natal, Western Cape, and North West provinces.

### 2.2. Sampling

Census-style sampling was used. All workers who met the eligibility criteria were invited to participate in the study. The pool of eligible workers was 334 employees. The response rate was calculated as follows: Response rate = actual number of responses/expected number of responses multiplied by 100.

### 2.3. Eligibility Criteria

To be eligible, workers had to be 18 years and over and had to have received at least one dose of the COVID-19 vaccine.

### 2.4. Pilot Study

A pilot study was conducted among 10 staff members. These staff members were removed from the greater survey dissemination. Minor corrections to skip patterns and required fields completion were made following the pilot.

### 2.5. Data Collection

Workers from the organisation were invited to complete a structured, self-administered online questionnaire (Supplementary Materials, File S1) using Microsoft Forms that explored determinants of COVID-19 vaccination, barriers and enablers to accessing vaccines, and worker perspectives on mandatory COVID-19 vaccine policies. Microsoft Forms is the preferred platform within the organisation in which this study was conducted; hence, the workers were familiar with the platform. The questionnaire consisted of 35 questions and had six sections that addressed the following areas: Section A: Demographics; Section B: Medical history; Section C: COVID-19 and the staff members’ individual experience; Section D Motivations for receiving COVID-19 vaccines; Section E: Enablers and barriers in accessing the vaccine; and Section F: Perspectives on mandatory workplace policies on COVID-19 vaccines. One question was open-ended, asking for perspectives on the mandatory vaccination policy, while the rest were closed-ended questions. Potential participants were emailed a link that provided access to the Microsoft Form that included the information sheet and consent form. All workers in the organisation have access to a laptop and/or other electronic device. Participants were able to complete the consent form and questionnaire at their convenience, including after hours. Participants who consented to participate were then sent a separate link to the questionnaire. To ensure confidentiality, participants’ personal details, such as email address or link to work laptops/tablets, were not linked to the questionnaire responses. The questionnaire was available from 11 September 2024 to 7 October 2024, with 4 reminders sent during this timeframe. The questionnaire was administered in English and took approximately 15 to 30 min to complete. There was no time limit to complete the questionnaire; however, participants only had a single attempt to complete the questionnaire.

### 2.6. Data Analysis

Data were analysed using IBM-SPSS version 29. Descriptive statistics (frequencies and percentages) were utilised to describe the data using categories. A Likert scale was utilised in the questionnaire to assess the reasons for COVID-19 vaccination. Open-ended questions were analysed by categorising themes for worker perspectives on the mandatory workplace policy and barriers and enablers to accessing COVID-19 vaccinations. Direct quotes are referenced in the Results section, including participants’ age and sex.

### 2.7. Ethics Approval

This study was conducted in accordance with the Declaration of Helsinki and approved by the University of Johannesburg, Faculty of Health Sciences Research Ethics Committee (REC-2949-2024 on 14 August 2024).

## 3. Results

Overall, 106 workers consented to participate, and of them, 91 workers responded to the survey, of which two were ineligible (did not receive any COVID-19 vaccine doses) and one initially consented but later declined participation, resulting in a total of 88 workers being included in this analysis. There was a 32% response rate. The mean age was 41 years, and 70% (*n* = 62) were female (Table 1). Almost three-quarters (*n* = 64) had either an undergraduate or a postgraduate education. Over one-third (35%, *n* = 31) reported a history of chronic illness, 12 of whom had hypertension.

More than three-quarters (77%, *n* = 68) of the respondents had been tested/investigated for COVID-19, and two-thirds (62%, *n* = 42) tested positive (Table 2). Approximately half of the respondents (48%, *n* = 42) were diagnosed with COVID-19.

Staff members received their first COVID-19 vaccination dose between 2020 and 2023, with over one-third (37%, *n* = 34) receiving their first dose in 2021. Thirty percent of staff were unable to remember the year of their first vaccine dose. Regarding the type of vaccine, almost two-thirds (65%, *n* = 57) received the Johnson & Johnson vaccine, and just under one-third (30%, *n* = 26) received the Pfizer vaccine. Four workers (5%) could not remember which vaccine they had received. The number of vaccine doses received ranged from one to four, with most workers (44%, *n* = 39) having received two doses. Almost two-thirds (63%, *n* = 55) reported receiving booster vaccine doses.

**Figure 1:**
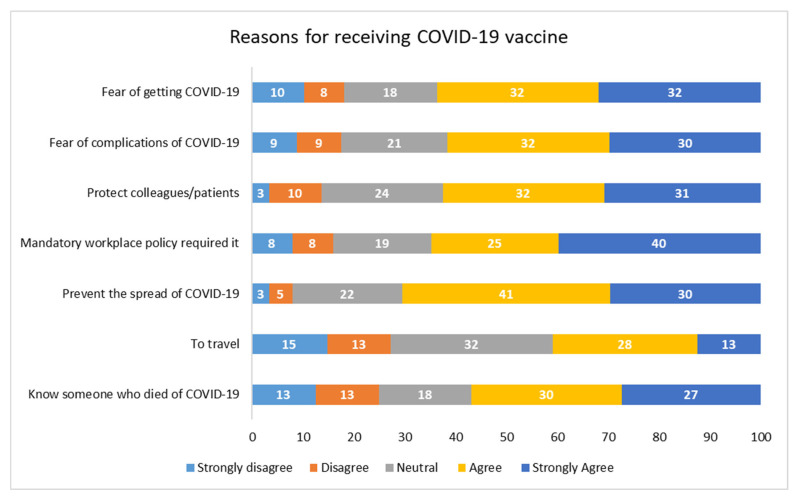
Reasons for receiving COVID-19 vaccine doses.

More than two-thirds (71%) of workers agreed/strongly agreed to be vaccinated against COVID-19 to prevent the spread of the virus (Figure 1). Other reasons for agreeing/strongly agreeing to receiving the COVID-19 vaccine included the mandatory workplace policy (65%), fear of contracting COVID-19 (64%), protecting colleagues and patients (63%). Travel was reported less often as a reason for being vaccinated, with only 41% agreeing/strongly agreeing with this reason. Neutral responses for all options ranged from 18% to 32%.

Other reasons reported by workers for receiving the COVID-19 vaccine included protecting family and community members, not wanting to infect others with COVID-19, consideration of age and chronic conditions, being afraid of reinfection with COVID-19, and for the benefit of herd immunity. Nearly three-quarters (74%) of the respondents were concerned about the adverse effects of the vaccine, and 66% reported being concerned or very concerned. Most workers received their vaccine doses at either a clinic (43%) or hospital (34%), and three-quarters reported that vaccines were easily or very easily accessible (Table 3). The reasons workers cited for easy access to the vaccine included that it was available at their workplace (42%), the vaccine roll-out site was close to their home/workplace (22%), and the vaccine was available at no cost (32%). Less than 10% of workers reported challenges in accessing the vaccine.

Factors that made it easy or very easy for workers to access vaccinations included vaccines being available at their place of employment, receiving expedited services as they were healthcare workers, and efficient processes at vaccine sites. Selected quotes are provided below:

“The vaccination sites were in my local town and a nearby town, with no waiting times, and professional and friendly staff.”

(41 years, male)

“It was fast and I did not have to wait long for it.”

(47 years, male)

“Firstly it was easy to get the Vaccine because there was transport taking workers to hospital to get the Vaccine. Secondly, I got it from my local clinic where am working.”

(51 years, female)

Multiple workers reported that the booking system and registration processes were easy and efficient and that this facilitated vaccination.

“Easy, registered online and went [for vaccination].”

(35 years, male)

“The system to book was easy to use and appointments were honoured.”

(53 years, female)

“Quick registration and quick access to vaccination.”

(55 years, female)

Challenges reported by workers in accessing the COVID-19 vaccine included long queues and having to travel far to receive the vaccine.

“At the time there was a huge influx of people getting the vaccine. I had to wake up extra early to drive to a hospital out of town in the hope that I would not have to wait all day. However, I still had to queue most of the day at the out-of-town hospital.”

(47 years, female)

Just under two-thirds of workers (63%) were supportive or very supportive of the mandatory COVID-19 vaccination policy implemented by the organisation, with only 15% reporting not being supportive or against the policy. The remaining 22% (*n* = 20) were either slightly or moderately supportive of the vaccination policy.

Reasons for supporting the mandatory workplace policy included feeling that vaccination would create a safer work environment, wanting to protect oneself and others from contracting COVID-19, receiving encouragement from their employer, senior staff, or colleagues to be vaccinated, and receiving adequate information about COVID-19 and vaccination (Table 4). Some workers also felt that the mandatory workplace policy demonstrated that the employer cared about employees’ well-being and health. Concerns reported by workers who were slightly or moderately supportive of the vaccine policy included limited knowledge on the COVID-19 vaccine, concern about side effects from the vaccine, and feeling coerced into being vaccinated. A few workers also felt that they should have been given a choice regarding vaccination. Reasons for being against or not supporting the mandatory COVID-19 vaccination policy included workers feeling as though they had no choice in being vaccinated or not and fearing that non-vaccination might impact their employment.

There was another pandemic, while 44% (*n* = 39) reported not being willing to be vaccinated.

## 4. Discussion

In this study, conducted in a workplace setting with a mandatory COVID-19 vaccine policy and high uptake among South African workers, the main reasons for COVID-19 vaccination included preventing the spread of COVID-19 (71%), fear of contracting COVID-19 (64%), protecting colleagues and patients (63%), and the mandatory vaccination policy (65%). Overall, vaccine access was generally easy and convenient for most workers. Almost two-thirds of workers said that they supported the mandatory COVID-19 vaccination policy, which was influenced by the desire to create a safer work environment, i.e., wanting to protect oneself and others from contracting COVID-19. Encouragingly, 58% of workers reported a willingness to vaccinate should there be another pandemic.

More than two-thirds of the workers (70%) agreed or strongly agreed to receive a COVID-19 vaccine to prevent the spread of COVID-19 in this study. This is consistent with other studies. Kozak and Nienhaus [14] in a study that included over 3400 health and social service providers in Germany observed that a key reason for COVID-19 vaccination among healthcare workers was largely related to protecting others (88%). Similarly, Stephanek et al. [15] found that among different occupational groups in the Czech Republic, including 3000 workers, common motives for receiving a COVID-19 vaccine included protecting family members (86%) and preventing the spread of COVID-19 (58%).

Since different factors influence the uptake of COVID-19 vaccines, there is a need for further understanding of different workplace environments and their policies and practices for vaccinations against other infectious diseases or emerging pandemics such as access to information, psychosocial support, and counselling to workers to educate them on diseases such as COVID-19 including the benefits of vaccination, creating a safer work environment and preventing morbidity and mortality among workers. Healthcare workers have a responsibility to take care of themselves and their clients/patients, and they could share information with them. Workplaces could serve as potential sources of accurate and reliable information.

In the current study, we found that approximately two-thirds (65%) of workers responded that their decision to receive the COVID-19 vaccine was influenced by the mandatory workplace vaccination policy. Other studies have also observed support for mandatory COVID-19 vaccination policies. In a study by Kozak and Nienhaus [14] with over 3000 healthcare workers in Germany, 58% of the respondents said that they would accept a mandatory workplace policy if it were implemented. In a systematic review and meta-analysis of healthcare workers’ attitudes towards COVID-19 vaccine mandates, including 57 studies that collectively had a sample of over 77,000 respondents, 64% of healthcare workers supported COVID-19 vaccine mandates for healthcare workers, while 50% supported vaccine mandates for the general population [11]

In a study by Kaufman et al. [16], 42% of healthcare worker respondents were supportive of a vaccine mandate, and, if forced by their employer, 57% of those who said that they did not plan to vaccinate reported that they would vaccinate if mandated to, indicating that mandatory workplace policies might increase uptake, particularly among those who might initially be reluctant to vaccinate. This is supported by Lee et al.’s [17] study findings of 91% uptake after implementation of the mandatory policy compared to 73% uptake among workers where there was no mandate.

As vaccine mandates are controversial, according to the WHO [18], vaccine mandates should be considered only after an opportunity has been given to be vaccinated voluntarily or when there is a rationale that societal or institutional objectives will not be met voluntarily. This is underscored by the fact that demonstrated efforts must be made to show and the WHO [18] confirms the need for transparency, fairness, objectivity, non-discrimination, and consultations with all parties in decision-making towards ethically justified policymaking. Considerations of mandatory vaccination workplace policies should include consultations with all relevant stakeholders, considering multiple factors to ensure that it is aligned with ethics, legal frameworks, occupational and public policy, available evidence, and fairness.

Reasons for supporting the mandatory vaccination policy in the study included feeling that vaccination would create a safer work environment, wanting to protect oneself and others from contracting COVID-19, and receiving consistent information on COVID-19. A systematic review of 15 studies by Okpani et al. [19] found that there was an increase in COVID-19 vaccination that coincided with vaccine mandates.

Only 15% (*n* = 13) of the respondents were against or not supportive of the mandatory COVID-19 vaccination policy. Reasons included dissatisfaction about not being given a choice about vaccination and the implications of not accepting vaccination for their employment and job security, which is understandable given the unemployment rate in South Africa, which was at 23% in 2022 [20].

Three-quarters of all workers (75%) in the study responded that they had very easy or easy access to vaccines, with more than one-third having access to vaccines where they work. A study by Myburgh et al. [21] conducted in South Africa and Zimbabwe among the general population identified access to vaccination sites as a motivator for vaccination. Additional motivators were proximity to the vaccine site, trust in the government, the conviction that vaccines can reduce the intensity of COVID-19, and previous experience from childhood vaccinations [21]. Findings from Kaufman et al. [16] highlighted the importance of vaccines being convenient and accessible for healthcare workers, such as the availability of vaccines on site and access to critical, clear information on COVID-19 and vaccinations to allow healthcare workers to recommend vaccines to their patients. Only 8% of the respondents reported difficulties in accessing COVID-19 vaccines in the study. In the study by Myburgh et al. [21], similar barriers such as long queues/waiting times and overcrowding at vaccine sites were reported.

Barriers and enablers to vaccination are also driven by context, and there should be further investigation into some of the aspects to allow for a more robust understanding across different contexts and settings. This will be critical to addressing future public health crises and allow for effective interventions to be implemented as part of workplace policies to minimise personal and structural barriers and facilitate access to vaccines could promote uptake such as vaccine sites close to workers’ homes/workplaces, vaccines being available at no cost, minimising long queues, booking systems, and receiving time off from work for vaccination to enable access. Where information on the safety and efficacy of vaccines is provided, intention to vaccinate is higher, and this is important for herd immunity as it requires very high levels of COVID-19 vaccine uptake [22].

One of the strengths of this study is that it was conducted in a workplace setting within one organisational project where there was a mandatory COVID-19 vaccination policy. Limitations include a low response rate, and this was likely related to the organisation undergoing a restructuring process (Section 189A) and staff being notified of retrenchment. We acknowledge the small sample size and lack of power in this study. This study took place in 2024, several years after the start of the COVID-19 pandemic and vaccination of workers; hence, recall bias among workers could have occurred. The first author, D.N., is employed in a senior position in the organisation, which could have influenced the responses from staff members. To minimise this impact, the letter sent to staff and the consent form explicitly reiterated the confidentiality of all responses, including the anonymity of the survey.

## 5. Conclusions

This study provides insights into reasons for receiving COVID-19 vaccines in a mandatory workplace setting, including preventing the spread of COVID-19, fear of contracting COVID-19, protecting colleagues and patients, and the mandatory vaccination policy. Workers were generally supportive of the mandatory workplace policy, and vaccine access was easy and convenient. Further research on motivations for receiving COVID-19 vaccination in mandatory workplace settings and perceptions of mandatory COVID-19 vaccination policies is needed, particularly to obtain insights that could aid decision-making and planning for future pandemics and occupational health policies within different contexts and settings.

## Figures and Tables

**Table 1:** Participant characteristics.

Characteristic	*N* (%) or Mean (Standard Deviation)
^1^ Age (years)	41 (9.98)
**Age categories (years)**	
18–39	35 (40)
40–49	22 (25)
≥50	13 (15)
Unknown	18 (20)
**Gender**	
Male	26 (30)
Female	62 (70)
**Level of education**	
Matric	24 (27)
Undergraduate	22 (25)
Postgraduate	42 (48)
**Marital status**	
Married	28 (32)
Unmarried	52 (59)
Prefer not to disclose	8 (9)
**Staff category**	
Lay	15 (17)
Clinical	19 (22)
Data staff	25 (28)
Multi-district support services	22 (25)
Other	7 (8)
**Smoker**	
Yes	10 (11)
No	78 (89)
**History of chronic illness**	
Yes	31 (35)
No	57 (65)
**Chronic illness**	
Diabetes mellitus	2 (2)
Hypertension	12 (14)
Respiratory disease	7 (8)
Diseases affecting the immune system None reported	9 (10) 58 (66)

^1^ 18 workers did not disclose their age.

**Table 2:** COVID-19 history.

Characteristic	*N* (%)
**Ever tested/investigated for COVID-19**	
Yes	68 (77)
No	20 (23)
**Ever tested positive for COVID-19**	
Yes	42 (62)
No	26 (38)
**Ever been diagnosed with COVID-19**	
Yes	42 (48)
No	46 (52)
^1^ ** Ever been hospitalised for COVID-19**	
Yes	8 (19)
No	34 (81)
**Family member ever had COVID-19**	
Yes	53 (60)
No	35 (40)

^1^ Ever hospitalised for COVID-19 is presented as a proportion of those diagnosed with COVID-19 (*n* = 42).

**Table 3:** COVID-19 vaccine access: Enablers and challenges.

Characteristic	*N* (%)
**^1^ Place where the vaccine was accessed:**	
Clinic	38 (43)
Hospital	30 (34)
Community venue	16 (18)
Private pharmacy	12 (14)
Other	3 (3)
**Ease of accessing the vaccine:**	
Very easy/easy	73 (83)
Neutral	8 (9)
Difficult/very difficult	7 (8)
**Factors that made it easy to access the vaccine:**	
The vaccine is available at the facility where I work	33 (42)
The vaccine roll-out site is close to my home or workplace	17 (22)
Available at no cost to me/for free	25 (32)
Other	3 (4)
**^2^ Challenges to accessing the vaccine:**	
Long queues	3 (3)
The roll-out site is far from my home	2 (2)

^1^ Multiple options were allowed therefore total percentage exceeds 100%. ^2^ Five participants reported challenges to accessing the vaccine.

**Table 4:** Quotes from workers regarding the mandatory workplace vaccination policy.

Quotes	Age and Sex of Worker
**Supportive/Very Supportive**	
“The COVID-19 vaccine is to protect myself and others, creating a safer work environment as well as protecting public health.”	28 years, female
“Our Company was supportive, especially in encouraging us to take the vaccine, making us understand the vitality of taking the vaccine. Even in the time of losing our colleagues they were supportive. And they checked on us even in time of isolation if were [we] came in contact with someone who had COVID [COVID-19].”	Unknown age, female
“A mandatory workplace policy signal [s] that employer cares about employees’ health and wellness. Vaccinated employees are protected against each other, their families and communities.”	67 years, male
**Slightly/Moderately Supportive**	
“There was insufficient knowledge about the vaccine itself.”	65 years, female
“As [an] employee, whatever concerns you may have had about [an] untested, unknown quotient rapid developed vaccination was triumphed by your need to remain employed, so even if you would have preferred to not take the vaccine, you were left with almost no choice, but to get [it] done. To the question if I would be willing to take a vaccine for another pandemic honestly I don’t know … but coerced [as] I had been for COVID19 I probably would have to.”	52 years, female
“The vaccination was compulsory I did not have a choice.”	Unknown age, female
“I felt that I am slightly coerced, since I had recently had a clotting of blood issue and there were issues around the vaccine and clotting.”	55 years, female
“I wish that each and every person was given the right to refuse or agree to get the vaccine.”	54 years, female
**Against/Not Supportive**	
“[It] felt like I did not have a say in whether I want to take the vaccine or not.”	58 years, male
“This was a forced measure with threats of loosing [losing] jobs, people’s rights against their health was [were] not considered.”	34 years, male
“You needed to decide to either vaccinate or lose your employment with the organization, so it wasn’t a good space to be in.”	28 years, female

## Data Availability

The data that support the findings of this study are available in a de-identified format from the corresponding author upon reasonable request.

## Supplementary Materials

The following supporting information can be downloaded at: https://www.mdpi.com/article/10.3390/ijerph22060929/s1. File S1: Questionnaire.

## Abbreviations

The following abbreviations are used in this manuscript:

COVID-19	Coronavirus Disease of 2019
DoH	Department of Health
HAST	HIV and AIDS/STI/TB
HIV/AIDS	Human Immunodeficiency Virus Acquired Immunodeficiency Syndrome
WHO	World Health Organisation
