# Supplementary material for: A Quantitative Study on Determinants of COVID-19 Vaccine Uptake in a Mandatory Vaccination Workplace Setting in South Africa
Source: Int J Environ Res Public Health. 2025 Jun 12;22(6):929. doi: 10.3390/ijerph22060929 (PMC12193624; doi:10.3390/ijerph22060929)
Supplement: Supplementary file 1 [file ijerph-22-00929-s001.zip › ijerph-3622438-supplementary.pdf]

APPENDIX A: Questionnaire

Determinants of COVID-19 Vaccine Uptake in a Mandatory Vaccination Workplace Setting in South Africa

Dear participants

I, Dhirisha Naidoo (Govender), am undertaking a research project to assess the Determinants of COVID-19 Vaccine Uptake in a Mandatory Vaccination Workplace Setting in South Africa. To this end I kindly request that you complete the following short questionnaire measuring the reasons for COVID-19 vaccination among clinical and non-clinical workers in an organization. It should take no longer than 30 minutes of your time. There are no right or wrong answers. Your participation in this survey is voluntary, information provided by you remains confidential, and would be used only by the research team for the purposes of the research.

Should you have any queries or comments regarding this survey, you are welcome to contact me on +2782 920 or e-mail me at dnaidoo@brhc.com or contact my supervisor on e-mail at btaderera@uj.ac.za

Firstly, we need to establish if you meet the section criteria to complete this survey.

Are you aged 18 years or older?

Yes	1
No	2

Have you had at least one vaccination for COVID-19?

Yes	1
No	2

Are you employed with BroadReach Health Development?

Yes	1
No	2

Do you give consent to participate in this study?

Yes	1
No	2

If you answered **No** to any of the above questions, please do not continue with the survey.

SECTION A: DEMOGRAPHICS

This section of the survey refers to your demographic information. Although we are aware of the sensitivity of the questions in this section, the information will allow us to compare groups

of respondents. Once again, we assure you that your response will remain anonymous. Your co-operation is appreciated.

1. How old are you?

Please enter response here	1
Prefer not to disclose	2

2. What is your gender?

Female	1
Male	2
Prefer not to disclose	3

3. What is your marital status?

Married	1
Unmarried	2
Prefer not to disclose	3

4. What is your highest level of education?

Matric	1
Undergraduate	2
Postgraduate	3

5. What category of staff do you fall under?

Lay (Coach Mpilo, Lay Counsellor, Linkage Officer, Community Mobiliser, Facility Case Managers)	1
Clinical (Nurse, Doctor, Pharmacist, Laboratory, Social worker)	2
Data staff (District Data information Officer, Data Quality and Use Manager, Data Capturer, SI/MER team)	3
Multi district support services (Human Resources, Finance, Contracts Grants Compliance, Administrators, Procurement)	4
Other (please specify):……..	5

6. How would you describe your position at work?

Mostly managerial	1
Mostly administrative	2
Mostly patient facing or interacting	3
Other (please specify):……..	4

SECTION B: MEDICAL HISTORY

This section asks you some questions about your medical history. This is important as we know that certain medical conditions put people at higher risk of severe disease and complications related to COVID-19.

7. Do you have any chronic diseases (such as Hypertension, Diabetes, Asthma) or any other condition that you take regular medication for)?

Yes	1
No	2

8. If yes, do you have any of the following chronic disease/s?

Diabetes Mellitus	Yes	No
Hypertension	1	2
Respiratory Diseases	1	2
Diseases affecting the immune system including autoimmune diseases	1	2

9. Have you ever smoked cigarettes?

Yes	1
No	2

If No, please skip to Question 11

10. If yes, how many cigarettes do you smoke daily?

1 – 5 a day	1
6 – 10 a day	2
More than 10 a day	3

SECTION C: COVID-19

This section asks you questions on whether you have been tested and/or diagnosed with COVID-19 before

11. Have you ever been investigated/tested for COVID-19?

Yes	1
No	2

If No, skip to Question 13

12. If yes, how many times have you been tested?

1-2 times	1
2-3 times	2
3-4 times	3
4-5 times	4
More than 5 times	5

13. Have you ever tested positive for COVID-19?

Yes	1
No	2

14. Have you ever been diagnosed with COVID-19?

Yes	1
No	2

If No, please skip to Question 16

15. Have you ever been hospitalized for COVID-19 and/or complications from COVID-19?

Yes	1
No	2

16. Has anyone in your family ever had COVID-19?

Yes	1
No	2

SECTION D: REASONS FOR COVID-19 VACCINATION

This section explores why you decided to receive a COVID-19 vaccine.

17. Which month and year was your first COVID-19 vaccination administered?

Month	1
Year	2
Cannot remember	3

18. Which type of vaccine did you receive?

Pfizer	1
Johnson and Johnson	2
Other, please specify?	3
Cannot remember	4

19. How many COVID-19 vaccine doses did you receive in total (excluding boosters)?

Insert number here?	1
Cannot remember	2

20. Did you receive any COVID-19 booster vaccines?

Yes	1

No	2
Cannot remember	3

21. To what extent do you agree or disagree with the reason/s for your decision to get one or more COVID-19 vaccine doses?

Fear of getting COVID-19	Strongly disagree	Disagree	Neutral	Agree	Strongly Agree
Fear of complications of COVID-19	1	2	3	4	5
To protect my family members	1	2	3	4	5
To protect my colleagues and/or patients	1	2	3	4	5
The mandatory workplace policy required me to get the vaccine	1	2	3	4	5
To prevent the spread of COVID-19	1	2	3	4	5
To travel	1	2	3	4	5
Know someone who died from COVID-19	1	2	3	4	5
Other, please specify?	1	2	3	4	5

22. Please indicate any other reasons for your decision?

23. Were you concerned about adverse effects of the vaccine?

Yes	1
No	2
Unsure	3

If no, skip question 24.

24. If yes, How concerned are you about the adverse effects of the vaccine?

Slightly concerned	1
Somewhat concerned	2
Concerned	3
Very concerned	4

SECTION E: ENABLERS AND BARRIERS TO COVID-19 VACCINE

We would like to understand any challenges or facilitators to getting the COVID-19 vaccine in this section.

25. Where did you access one or more COVID-19 vaccine doses? (Please select all options that apply)

Local Clinic	1

Hospital	2
Community venue	3
General Practitioner (GP)	4
Private Pharmacy	5
Other, please specify.	6

26. How easy was it for you to access the COVID-19 vaccine.

Very easy	1
Easy	2
Neutral	3
Difficult	4
Very difficult	5

27. What made it easy or difficult for you to get the vaccine? Please describe.

28. Were there any factors that made it easy for you to get the COVID-19 vaccine?

Yes	1
No	2

If No, skip to Question 25

29. If yes, what were some of the factors that made it easy for you to get the vaccine? (Please select all that apply)

Vaccine available at facility where I work	1
Vaccine roll out site close was close to my home or workplace	2
It was available at no cost to me / for free	3
Other, please specify.	4

30. Did you experience any challenges in getting the vaccine?

Yes	1
No	2

If No, skip to Question 27

31. What challenges or barriers did you experience in getting the COVID-19 vaccine? (Please select all that apply)

Vaccine unavailable/ no stock of vaccine at facility/site	1
I was asked to return for the vaccine on a different date or referred to an alternate site	2
Long waiting queues	3
Vaccine roll-out site was far from my home or workplace	4
Other, please specify?	5

SECTION F: WORKPLACE POLICIES

We would like to understand your opinion on the mandatory workplace policy. Please remember that this survey is anonymous, and your response will not be used in any way against you or affect your employment at the organization.

32. Which of the following best describes how you felt about mandatory workplace COVID-19 vaccine policy? (This refers to the company requirement of all staff being vaccinated against COVID-19)

Very supportive	1
Supportive	2
Moderately supportive	3
Slightly supportive	4
Against Not at all supportive	5

33. Based on your response above, please explain?

34. Would you be willing to take a vaccine again if there was another pandemic?

Yes	1
No	2

Thank you very much for your time. This concludes the survey.
